# Malaria Transmission Pattern in an Area Selected for Clinical Trials in the Sudanian Area of Senegal (West Africa)

**DOI:** 10.1155/2013/907375

**Published:** 2013-02-05

**Authors:** El Hadji Amadou Niang, Aissatou Touré, El Hadji Malick Ngom, Lassana Konaté, Ousmane Faye, Mawlouth Diallo, Ibrahima Dia

**Affiliations:** ^1^Unité d'Entomologie Médicale, Institut Pasteur de Dakar, BP 220, Dakar, Senegal; ^2^Laboratoire d'Ecologie Vectorielle et Parasitaire, Département de Biologie Animale, Faculté des Sciences et Techniques, Université Cheikh Anta Diop de Dakar, BP 5005, Dakar, Senegal; ^3^Unité d'Immunologie, Institut Pasteur de Dakar, BP 220, Dakar, Senegal

## Abstract

Malaria transmission pattern was studied in 3 villages (Toubanding, Daga Ndoup, and Keur Samba Guèye) situated within an area selected for clinical trials. The study was conducted in the rainy season from July to December 2011. The main objective of this work was to gather baseline data on malaria transmission intensity and other entomological parameters before the advent of clinical trials. Mosquitoes were collected by Human-Landing Collections (HLCs) and by pyrethrum spray catches (PSCs). Five anopheline species were collected, namely, *An. arabiensis*, *An. gambiae*, *An. funestus*, *An. pharoensis*, and *An. rufipes*, giving a heterogeneous distribution within the study area. The populations dynamics of the vectors varied temporarily in each village depending on the pattern of the rainy season. Transmission intensity estimated by the entomological inoculation rate (EIR) was measured in each of the three villages with the variations linked to the microecological differences between the villages. Measurements were calculated for August, September, and October and were found to vary between 4 and 30 infected bites per person over the study period with a peak intensity observed in September. These results indicate that epidemiological field trials on malaria could be conducted in this area on the basis of the differences observed with transmission intensity, micro-ecological variations, and the objectives of the trials.

## 1. Background

Malaria continues to be a major public health problem throughout the world despite more than a century of study, especially in Africa where 90% of the global cases are recorded. The situation is worsening due to the spread of drug resistant parasites strains, spread of insecticide resistance in the vector populations, and poor economic status of endemic populations [1]. To alleviate the problem, an integrated approach against both the parasites and vectors for an effective control is necessary. 

Over the last five years, considerable efforts have been made to control malaria in many countries around the world (especially in Sub-Saharan Africa) using strategic measures with available tools. This has led to the decline in malaria transmission in many parts of Africa [2, 3]. These changes are as a result of an extensive use of long-lasting insecticidal nets (LLINs) and improved malaria diagnosis and treatment. However, despite these significant progresses, malaria remains an acute problem killing 800000 people each year, mostly children under five years living in Sub-Saharan Africa [1]. The situation is particularly worrying with the increase in poverty for sustainability and more specifically as different models predict a loss of immunity of the populations with the current interventions [4, 5]. The rebound effect and age shift of malaria morbidity associated with an increasing susceptibility of older children and adults as seen in many places after the introduction of control strategies also help to sustain this hypothesis [6]. Change in behaviour of the vector populations from being endophilic to exophilic also makes the populations vulnerable [7]. The need for efficient and effective sustainable strategies including curative treatments and vaccines for malaria control is therefore eminent. The evaluation of such strategies requires detailed information on the epidemiology of malaria and the vector populations. An ideal indicator of malaria risk is the entomological inoculation rate (EIR), a parameter that relates both the behaviour and human-biting activity of the anopheline vectors and the risk to humans of malaria infection. Risk of exposure of human to infectious bites of vectors is not uniform in any geographical setting even within limited distances in an area. The variations in the abundance and dispersal of mosquitoes occur spatially and temporally in a given area and these variations can impact on the level of malaria transmission [8]. Thus, the evaluation of interventions under conditions of natural transmission requires that testing sites should be identified and characterized with baseline information derived before the implementation of the interventions.

Indeed, it is necessary to gather precise information and make a close follow up of variations in malaria transmission in an area identified for interventions to enable correct interpretations of malaria parameters such as parasitaemia, morbidity, and associated immune responses in relation to efficacy [9].

This study was undertaken within the European and Developing Countries Clinical Trials Partnership (EDCTP) framework aimed at characterizing selected study sites for clinical trials in the Sudanian bioclimatic area of Senegal. Temporal and seasonal variations in species composition, density, biting behaviour, and intensity of malaria transmission rates and ecological parameters are the key indicators that have been studied. 

## 2. Materials and Methods

### 2.1. Study Sites

**Figure 1:**
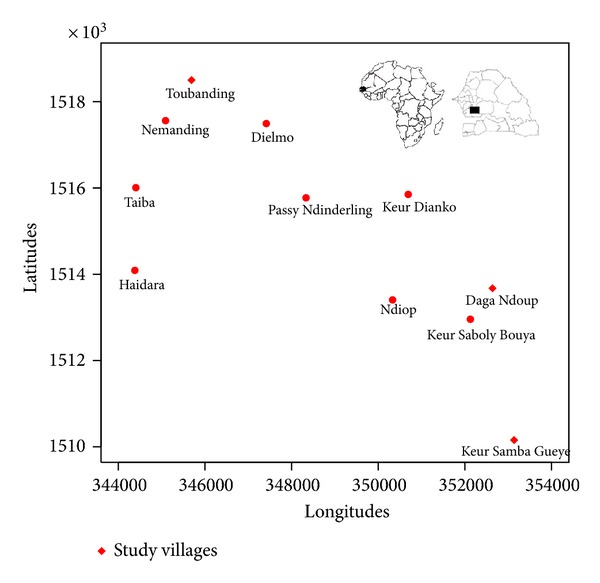
Map of the study area showing the three study villages.

The study was conducted in the rural community of Toubacouta in the Sine Saloum region. Nine villages were identified in the area and 3 (Daga Ndoup, Keur Samba Guèye, and Toubanding) were selected for entomological monitoring from July to December 2011 (Figure 1). The GPS coordinates for each of the 9 villages were recorded and the water network system identified as well as socioecological features like landscape, agricultural practices, access to health facilities, and vector control measures. Epidemiological and demographic data were also collected and parameters include age variations, inclusion rates. The selected villages are situated around the field research stations of Dielmo and Ndiop where extensive research on malaria has previously been conducted [10, 11]. Nema river passes through Toubanding, whereas the nearest water body (a pool) around Keur Samba Guèye is situated 1 km away with temporary puddles within the village of Daga Ndoup during the rainy season. The climate is Sudan-type savanna in this region with a rainy season that lasts from June to mid-October. The recorded monthly rainfall derived from Tropical Rainfall Measuring Mission (TRMM) data within the study area was 101, 134, 162, 153, and 118 mm from July to November 2011, respectively. Farming activities are concentrated mainly on food and cash crops (maize, millet, groundnuts, and vegetables). Trade and rearing of domestic animals like cows, sheep, goats, and chicken are also common practices of the people. Houses are of traditional types with mud walls and thatched or corrugated iron roofs. Ethical approval for this study was obtained from the Senegalese National Ethics Committee.

### 2.2. Mosquito Sampling and Field Processing

Entomological surveys were conducted using two classical methods: all nights Human-Landing Collections (HLCs) from two selected sentinel houses in each village (indoors and outdoors for two consecutive nights each month) and pyrethrum spray collections (PSCs) in 10 randomly selected rooms in each village. After collection, mosquitoes were sorted, counted, and morphologically identified to species [12]. A proportion of unfed females from each species were dissected to extract ovaries and to determine parity by observing the degree of coiling of ovarian tracheoles [13]. The blood meals from freshly fed females collected by PSC were squashed onto Whatman filter paper and dried for host source identification. All the mosquito samples collected were stored individually in numbered vials with desiccant until laboratory processing.

### 2.3. Laboratory Processing

The origin of blood meals from freshly fed indoor resting females collected after pyrethrum spray collections was identified as human, bovine, ovine, and horse using an Enzyme-Linked Immunosorbent Assay (ELISA) from Beier et al. procedure [14]. The heads and thoraces of all anopheline females were tested by ELISA for the detection of *Plasmodium falciparum* circumsporozoite protein (CSP) using Wirtz et al. procedure [15]. For each month, a random sample of 30 females belonging to the *An. gambiae *complex was identified to species and molecular forms levels by the molecular method described by Fanello et al. [16]. All CSP positive *An. gambiae *s.l. mosquitoes were also analysed by the same molecular method. 

### 2.4. Data Analysis

The human-biting rate (HBR) was defined for each species collected as the ratio of the total number collected to the total person-nights for the collection period. The endophagous rate was defined as the proportion of mosquitoes captured indoors against the total of both indoors and outdoors collections from HLC. The circumsporozoite rate was calculated as the proportion of total numbers of mosquitoes collected found to contain the *Plasmodium falciparum* CS protein. The anthropophilic rate was calculated as the proportion found with human blood out of the total analysed. The entomological inoculation rate (EIR) was calculated as the product of the human-biting rate (HBR) and the CSP rate of mosquitoes collected from night catches. All these parameters were computed and analysed using the free software R-gui version 2.15.1.

## 3. Results

### 3.1. Anopheline Collections

A total of 468 *Anopheles* specimens were collected from July to December 2011 by HLC and the composition includes mainly *An. gambiae* s.l., *An. funestus*, and* An. pharoensis*. *An. gambiae* s.l. was the predominant species in the three villages (Table 1). *An. funestus* was also collected in the three villages but its abundance was the highest only in Toubanding village (20%). In Daga Ndoup and Keur Samba Guèye, 1.7% and 3% were represented by *An. funestus*. *An. pharoensis* was less represented in the collections and was found only in Keur Samba Guèye and Toubanding villages.

Collections in human dwellings by PSC have yielded 748 anopheline females (Table 1). *An. gambiae* was found to be the predominant species in all three villages followed by *An. funestus* collected in Toubanding mainly and to a lesser extent in Daga Ndoup. Although we did not collect *An. rufipes* by HLC, a good number was collected in Keur Samba Guèye village. *An. pharoensis*, on the other hand, was not found resting in dwellings in all three villages.

Out of the 468 *An. gambiae* s.l. females collected by HLC, 168 (37 in Daga Ndoup, 47 in Keur Samba Guèye and 84 in Toubanding) were analysed using the PCR-RFLP. In all 3 villages, *An. arabiensis* was found to be the predominant species comprising 73% in Daga Ndoup, 91.5% in Keur Samba Guèye, and 65.5% in Toubanding. 

### 3.2. Biting Cycles

The mean number of bites per person per night (bpn) was significantly different for *An. gambiae* between the three villages (*F*_2,33_ = 15.8, *P* < 0.001) and for *An. funestus* (*F*_2,33_ = 4.2, *P* = 0.02), with the highest biting rates being observed in Toubanding village. For *An. pharoensis*, no significant difference was observed in biting rates between the 3 villages (*F*_2,33_ = 2.9, *P* = 0.07).

**Figure 2:**
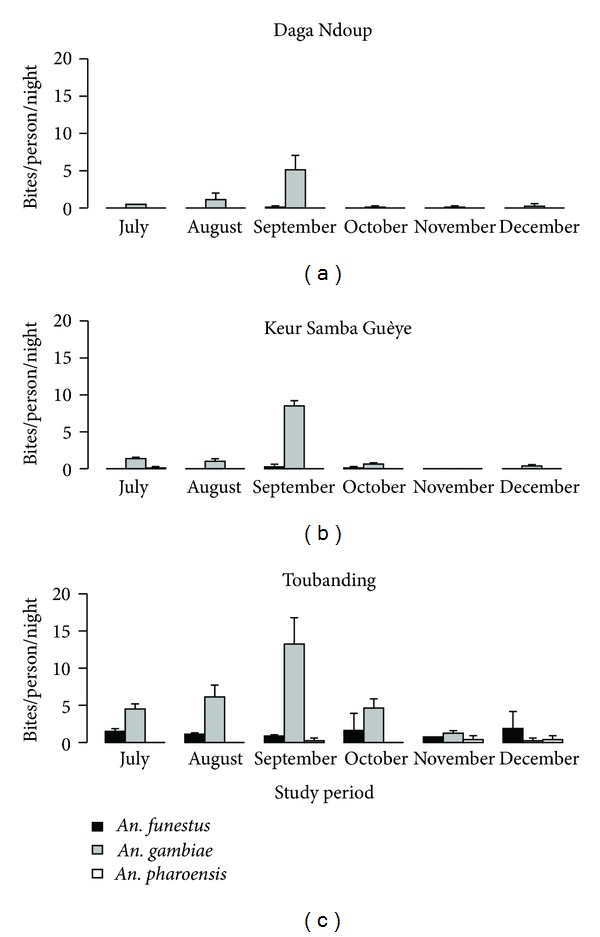
Temporal dynamics of *An. gambiae*, *An. funestus*, and *An. pharoensis *in Toubanding, Keur Samba Guèye and Daga Ndoup villages from July to December 2011.

In each of the three villages, the HBR peaked in September for *An. gambiae* (Figure 2). *An. funestus* females were collected only in Toubanding village throughout the study period with the highest density observed in December (Figure 2). For Daga Ndoup, this species was only present in September (0.12 bpn) and in September (0.25) and October (0.12 bpn) for Keur Samba Guèye village.

The biting rates for *An. pharoensis* females were generally very low with Keur Samba Guèye recording 0.12 bpn for July only and Toubanding, 0.25 bpn for September, and 0.37 bpn for both November and December.

### 3.3. Host-Seeking Behaviour

Overall, in Toubanding village, 27.4% of *An. funestus* and 47.9% of *An. gambiae* captured by HLC were collected indoors. These proportions were significantly different (*χ* ^2^ = 7.6, df = 1, and *P* = 0.006). The proportions of total *An. gambiae* collected by HLC indoors were not significantly different between the three villages, (*χ* ^2^ = 1.9, df = 2, and *P* = 0.38). One female *An. funestus* was collected outdoors in Daga Ndoup and 1 female *An. pharoensis* indoors in Keur Samba Guèye.

A total of 413 blood meals from blood fed females from indoor resting mosquitoes (385 *An. gambiae* and 28 *An. funestus*) were collected by PSC and tested by ELISA (Table 2). The proportion of human blood meals was 62.6% in Daga Ndoup, 65.2% in Keur Samba Guèye, and 48.3% in Toubanding and there was no significant difference between the three villages (*χ* ^2^ = 6.2, df = 2, and *P* = 0.05). Mixed blood meals were observed for *An. gambiae* in Daga Ndoup (8%), Keur Samba, Guèye (8.3%), and in Toubanding (13.9%). Other sources of blood meal were from cattle (Bovine) and Equine for Daga Ndoup and Keur Samba Guèye and from Ovine for Toubanding. 

The proportion of mixed blood meals was 7.4% in Toubanding for *An. funestus*. Overall, 24% were from human source, 72% Bovine, and 4% Equine (Table 2). In Daga Ndoup, only one *An. funestus* fed on human was collected. 

### 3.4. Parity Rates

A total of 39 females *An. gambiae *s.l. were dissected for parity from Daga Ndoup, 51 from Keur Samba Guèye, and 206 from Toubanding and the results are presented in Table 3. The parity rates were significantly different for *An. gambiae* between the three villages (*χ* ^2^ = 9.5, df = 2, and *P* = 0.009). Parity rate was higher in Keur Samba Guèye, compared to the other two villages. Parity rate was 78% for *An. funestus* in Toubanding village. There is a significant difference with *An. gambiae* females collected in the same village (*χ* ^2^ = 8.6, df = 1, and *P* = 0.003).

### 3.5. Circumsporozoite (CSP) and Entomological Inoculation Rates (EIR)

CSP ELISA to detect *P. falciparum* circumsporozoite antigen was conducted on all 468 *Anopheles *specimens collected in the three villages. A confirmatory test was done on all specimens giving positive ELISA results. For the *An. gambiae *s.l. species collected in the 3 villages (Toubanding, Keur Samba Guèye, and Daga Ndoup), the sporozoite rates were, respectively, 2.50% (CI 95% = 1.15–5.35), 1.05% (CI 95% = 0.19–5.72), and 1.72% (CI 95% = 0.3–9.13). In the *An. funestus* samples collected in Toubanding, 3.23% (CI 95% = 0.89–11.03) were positive (Table 4). The differences were not statistically significant for *An. gambiae *s.l. circumsporozoite protein rates between the three villages (*χ* ^2^ = 0.7, df = 2, and *P* = 0.68).

The entomological inoculation rate (EIRs) for this area was estimated at 30 infective bites per person during the study period in Toubanding, 4 infective bites per person in Daga Ndoup and Keur Samba Guèye. In Daga Ndoup and Keur Samba Guèye,* An. gambiae* s.l. was mainly responsible for the transmission and it was concentrated in September, whilst in Toubanding both *An. funestus* and *An. gambiae* were responsible for transmission for 3 months of the season (August, September, and October).

## 4. Discussion

During this study, five out of 20 anopheline species described in Senegal [17] as well as the molecular forms of *An. gambiae* s.s. (M and S) were recorded. The predominant species within the *Anopheles gambiae* complex from the collections in this area is *An. arabiensis*. This observation contrasts with the recent records in the area that show that *An*. *gambiae* s.s. represents about 80% of the species of *An. gambiae* complex collected [6]. Such differences could be due to differences in microgeographic ecological characteristics within the study area. 

The highest densities were observed in Toubanding village only. This observation again could be related to the ecological differences between our study villages. This discrepancy is sustained by the fact that other vector species were present. In Daga Ndoup and Keur Samba Guèye, mosquito-breeding sites are rain dependent with pools and puddles indiscriminately scattered around the village for short periods only. Toubanding, on the other hand, is located near a small stream that permits the development of anopheline breeding sites for longer periods. In Dielmo village situated 1.5 km apart, this stream permits the persistence of anopheline breeding sites all year round [18]. This can explain the presence of *An. funestus* and *An. gambiae* throughout the duration of the study period. The populations' dynamics of these species have similar characteristics to what is usually observed in Sahelian zones [12, 19]. Fluctuations in populations' densities of *An. gambiae* are related to rainfall pattern as is generally found in the other bioclimatic areas of Senegal [20–22].

Despite the limited distances between the selected villages, there was an 8-fold variation between Toubanding compared to the other two villages in terms of transmission potential. This is probably mainly due to the heterogeneity on anopheline vectors as observed. Such an observation was already assessed between the two most studied villages in the study area, namely, Dielmo and Ndiop [10, 11]. The transmission was seasonal, occurring for only one-to-three months of the year depending on the village locations. In Daga Ndoup and Keur Samba Guèye, malaria transmission is observed only in September, whilst in Toubanding, transmission occurs from August to October with a peak period observed in September. Daga Ndoup and Keur Samba Guèye villages have temporal breeding sites and Toubanding has what is a more permanent breeding ground for vectors throughout the year. However, it is important to note that despite the existence of this stream in Toubanding, malaria transmission does not proceed beyond October. Even if there is a transmission after October, it will be at low levels below the sensitivity of the method used to detect transmission. This is contrary to the existence of perennial transmission in the village of Dielmo thanks to the presence of the Nema river that permits and supports the persistence of anopheline breeding sites all year round [6].

In Toubanding village, which showed the highest densities of *An. gambiae* populations, compared with Daga Ndoup and Keur Samba Guèye, the anthropophilic rate is very low. A similar scenario was observed for the same species in Burkina Faso [23], in Burundi [24], and recently in *An. funestus* populations from northern Senegal [22]. Given the comparable levels of infestations observed, the highest transmission estimated in Toubanding in the case of *An. gambiae* is probably due to higher densities observed for this vector species.

It is noteworthy to mention the main reasons for low levels of transmission in Keur Samba Guèye and Daga Ndoup, which has similar transmission levels considering the fact that local divergences are obvious with the presence of the Nema river near Toubanding village. The results obtained for Daga Ndoup and Keur Samba Guèye could be extrapolated to all other villages in the area with rain-dependent breeding sites. However, one should be cautious of the fact that other local ecological features or interannual variations in rainfall could lead to differences between villages as was already observed elsewhere [10, 11, 25, 26]. 

Taking into account our collated entomological results and the specific objectives of the study as set out initially, epidemiological field trials can be conducted in this area. In this process, the timeline should target the peak transmission period, which is September, especially for vaccine in order to achieve peak antibody response or peak infection rate with increasing transmission.

## Figures and Tables

**Table 1:** Number of anopheline species collected by HLC and PSC in Daga Ndoup, Keur Samba Guèye, and Toubanding from July to December 2011.

Mosquito species	Daga Ndoup			Keur Samba Guèye			Toubanding			
	HLC		PSC	HLC		PSC	HLC		PSC	Total
	In	Out	In	In	Out	In	In	Out	In	
*An. funestus *	0	1	2	3	0	0	17	45	54	122
*An. gambiae *	22	36	403	45	50	118	115	125	170	1084
*An. pharoensis *	0	0	0	1	0	0	6	2	0	9
*An. rufipes *	0	0	0	0	0	1	0	0	0	1

Total	22	37	405	49	50	119	138	172	224	1216

**Table 2:** Proportions of *An. gambiae* and *An. funestus *fed on each vertebrate host in Daga Ndoup, Keur Samba Guèye, and Toubanding among resting mosquitoes.

Species	Villages	Number	Vertebrate hosts (%)				Mixed
		identified	Human	Bovine	Ovine	Equine	
	Daga Ndoup	212	62.6 [55.6–69.1]	27.2 [21.4–33.8]	0 —	10.3 [6.7–15.3]	8 [5.1–12.5]
*An. gambiae *	Keur Samba Guèye	72	65.2 [53.1–75.5]	31.8 [21.8–43.8]	0 —	3 [0.8–10.4]	8.3 [3.9–17]
	Toubanding	101	48.3 [38.1–58.6]	42.5 [32.7–753]	1.1 [0.2–6.2]	8 [3.9–15.7]	13.9 [8.4–21.9]

	Daga Ndoup	1	100	0	0	0	0
*An. funestus *	Keur Samba Guèye	—	—	—	—	—	—
	Toubanding	27	24 [11.5–43.4]	72 [52.4–85.7]	0 —	4 [0.7–19.5]	7.4 [2.1–23.4]

[  ]: 95% confidence interval.

**Table 3:** Parity rates of *An. gambiae* and *An. funestus* in Daga Ndoup, Keur Samba Guèye, and Toubanding during the six surveys.

Species	Villages	Dissected	Parous	PR [95% CI]
	Daga Ndoup	39	15	38.5 [24.9–54.1]
*An. gambiae *	Keur Samba Guèye	51	36	70.6 [57–81.3]
	Toubanding	156	83	53.2 [45.4–60.9]

	Daga Ndoup	—	—	—
*An. funestus *	Keur Samba Guèye	—	—	—
	Toubanding	50	39	78 [64.8–87.2]

PR: parity rate in percentage.

[  ]: 95% confidence interval.

**Table 4:** Mean infection rate calculated by ELISA for *P. falciparum* for *An. gambiae*, *An. funestus*, and *An. pharoensis* in Daga Ndoup, Keur Samba Guèye, and Toubanding.

Villages	Species	HBR (b/p/n)	CSPR (%)	EIR (Ib/p/n)
	*An. funestus *	0.02	0	0
Daga Ndoup	*An. gambiae *	1.21	1.72 (0.3–9.13)	0.02 (0.004–0.11)
	*An. pharoensis *	0	0	0

	*An. funestus *	0.06	0	0
Keur Samba Guèye	*An. gambiae *	1.98	1.05 (0.19–5.72)	0.02 (0.004–0.11)
	*An. pharoensis *	0.02	0	0

	*An. funestus *	1.29	3.23 (0.89–11.03)	0.04 (0.01–0.14)
Toubanding	*An. gambiae *	5	2.50 (1.15–5.35)	0.13 (0.006–0.27)
	*An. pharoensis *	0.17	0	0

HBR: human-biting rate.

CSPR: circumsporozoite rate.

EIR: entomological inoculation rate.

Ib/p/n: infective bites per person per night.

(  ): 95% confidence interval.
